# The performance of COVID-19 Surveillance System as timely containment strategy in Western Oromia, Ethiopia

**DOI:** 10.1186/s12889-021-12380-6

**Published:** 2021-12-18

**Authors:** Afework Tamiru, Bikila Regassa, Tamirat Alemu, Zenebu Begna

**Affiliations:** 1 Department of Public Health, Institute of Health Sciences, Wollega University, Nekemte, Ethiopia; 2 Department of public health, college of medicine and health sciences, Ambo University, Ambo, Ethiopia

**Keywords:** Evaluation, Surveillance, Suspect, West Ethiopia

## Abstract

**Background:**

COVID-19 has been swiftly spreading throughout the world ever since it emerged in Wuhan, China, in late December 2019. Case detection and contact identification remain the key surveillance objectives for effective containment of the pandemic. This study was aimed at assessing performance of surveillance in early containment of COVID 19 in Western Oromia, Ethiopia.

**Methods:**

A cross-sectional study was conducted from August 1 to September 30, 2020, in the 7 kebeles of Nekemte and 2 kebeles of Shambu Town. Residents who lived there for at least the past six months were considered eligible for this study. Data were collected from community and health system at different levels using semi structured questionnaire and checklist, respectively. Participants’ health facility usage (dependent variable) and perceived risk, awareness, Socioeconomic Status, and practices (independent variable) were assessed. Bivariable analysis was computed to test the presence of an association between dependent and independent variables. Independent predictors were identified on multivariable logistic regression using a p-value of (<0.05) significance level. We have checked the model goodness of fit test by Hosmer-lemeshow test.

**Results:**

One hundred seventy-nine (41%) of the participants believe that they have a high risk of contracting COVID-19 and 127 (29%) of them reported they have been visited by health extension worker. One hundred ninety-seven (45.2%) reported that they were not using health facilities for routine services during this pandemic. Except one hospital, all health facilities (92%) were using updated case definition. Three (33%) of the assessed health posts didn’t have community volunteers. On multivariable logistic regression analysis, the source of income AOR=0.30, 95% CI (0.11, 0.86), perceived level of risk AOR=3.42, 95% CI (2.04, 5.7) and not visited by health extension workers AOR=0.46, 95% CI (0.29, 0.74) were found to be independent predictors of not using health facilities during this pandemic.

**Conclusion:**

Event based surveillance, both at community and health facility level, was not performing optimally in identifying potential suspects. Therefore, for effective early containment of epidemic, it is critical to strengthen event based surveillance and make use of surveillance data for tailored intervention in settings where mass testing is not feasible.

## Background

On December 31, 2019, China reported the Outbreak of a novel strain of coronavirus. WHO named the new coronavirus, SARS-CoV2, and the disease: COVID-19 (Corona Virus Disease 2019) that later come to cause pandemic [1–4]. However, the source of infection is not confirmed, the isolation of the virus from the environmental samples taken from the Huanan seafood market (the common point for the first few cases) considered suggestive of wild animal sources that have been on sale at the seafood market [4, 5]. Person-to-person transmission is thought to occur among close contacts mainly via respiratory droplets produced when an infected person coughs or sneezes [6]. Individuals can also be infected from and touching surfaces contaminated with the virus and touching their face (e.g., eyes, nose, and mouth). Current information suggests that people are, generally, susceptible to COVID-19 [7]. Then, the transmission gets its next breakthrough as the disease spread among human beings (person to person transmission).

As of May 17, 2020, there were 4,529, 027 cases and 307, 565 deaths reported globally. America was the most affected country with 43.4% of global cases and 38.6% of global deaths from COVID-19. From the Africa region 58, 663 cases and 1, 710 deaths were reported. The leading country in Africa was South Africa 14, 355 cases and 261 deaths [8]. The Pandemic was first confirmed in Ethiopia on March 13, 2020, 2 months after its occurrence in Wuhan, China, and a month after the first case seen in Africa, Egypt. In Ethiopia, as of May 18, 2020, 352 cases and 5 deaths were reported [9]. Mitigation measures in such a situation should focus on early case detection and quarantine of their contacts. Surveillance plays a critical function in this regard.

The first response to the epidemic in Ethiopia was to set up a screening center at the point of entry. If the entering individual's temperature surpasses 38 degrees Celsius, he or she will be quarantined for 14 days, and if symptoms emerge, a sample will be obtained and sent to South Africa for testing. Because this approach relies solely on thermal screening, it is unable to detect those who have been exposed, and the majority of cases are likely to go unnoticed. Thermal scanning at the borders successfully detected roughly 25% of imported confirmed cases of the H1N1-2009 pandemic, largely at the airport, according to lessons learned from Singapore's HINI-2009 public health measures [10]. In Ethiopia, a 48-year-old Japanese index case passed thermal scanning on March 4, 2020, and was confirmed as positive for COVID 19 on March 12, 2020, following multiple contacts with community and commercial sites [11].

The emergence of major cases without verified contact history and cases from a dead body were features of the early pandemic situation in Ethiopia. Furthermore, cases are rising due to the lack of travel restrictions across the country, especially to and from Addis Ababa, Ethiopia's capital and epicenter of the epidemic. The number of imported cases rapidly increased. Later, the parliament ratified a state of emergency that restricts individual and social activities, including school closures.

One of the public health responses of Ethiopia was establishing COVID-19 surveillance throughout the country. The existing Integrated Disease Surveillance and Response (IDSR) was enhanced to identify all cases. Public health surveillance is the ongoing, systematic collection, analysis, interpretation, and dissemination of data regarding a health-related event for use in public health action to reduce morbidity and mortality and to improve health [12]. The system is intended to track routine and ad hoc data both inside and outside the health system and utilize it to identify public health risks. The two main components of a national surveillance system are indicator-based surveillance (IBS), which has an established case definition and performance indicator, and event-based surveillance that complement IBS in scanning the environment to detect potentials risks [13]. Event based surveillance is designed to recognize events and emerging public health threats that might otherwise go undetected by the surveillance system.

**Fig. 1:**
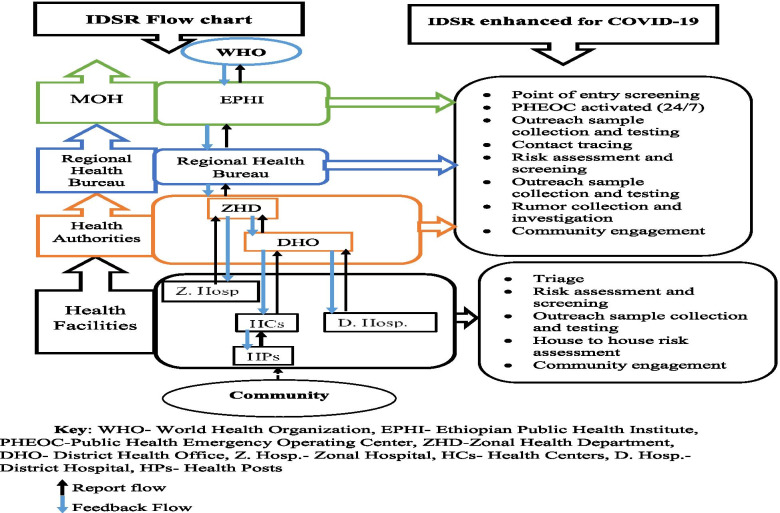
IDSR data and report flowchart with what has been enhanced for COVID-19

The health system in Ethiopia consists of primary level health care (composed of 5 health posts, 1 health center, and a district hospital), secondary level health care (1 general hospital), tertiary level health care (1 specialized hospital). The coordinating office was organized following the administration structure, i.e. Woreda health office, zone health department, regional health bureau and federal ministry of health. The IDSR, which consists of 7 weekly and 14 immediately reportable diseases, was established based on these structures and managed by the public Health Emergency Management unit available at each level (Fig. 1). The initial phase in COVID 19 surveillance is to identify suspects through enhanced rumor gathering, which includes rumor from various service locations, screening sites (Airport and land crossings), community-based surveillance, health-care triage centers and other sources. A toll-free team established at the national and regional level collects rumors from community and service points. The Rapid Response Team (RRT) will be responsible for verifying and investigating the rumor. If the reported rumor meets the criteria for a suspect case, the RRT will proceed with the standard suspect management procedures.

The key reporters in community-based surveillance (CBS) are community members who notice and report cases that would not otherwise be reported to healthcare facilities. At the local level, identified community volunteers will collaborate with Health Extension Workers (HEW) to enhance the CBS. To identify suspects at house hold level, a team of community volunteers with one HEW is required to visit at least 60 houses per day in agrarian rural areas, 40 households per day in pastoralist areas, and 120 families per day in per-urban and urban areas. The team must revisit the households twice a month. HEWs are responsible to coordinate overall activities of CBS at the kebele level (the lowest administration level with up to 5000 households). It will be investigated within 24 hours of receiving a community alert or identifying a suspect during a house to house visit.

There will be a pre-triage arrangement at every health facility where patients will be assessed for meeting recognized suspect criteria, such as (a patient with acute respiratory illness (fever/recent history of fever and or at least one sign and symptom of respiratory disease (e.g. cough, shortness of breath). Additionally, a history of travel to or residence in a country/area or territory reporting local transmission of COVID 19 in the last 14 days before symptom onset OR a patient with an acute respiratory illness AND having been in contact with a confirmed or probable COVID-19 case 1 in the last 14 days before the onset of symptom). An individual suspected to have COVID-19 in a pre-triage area will be directed to the triage designated area for further assessment and decision on referral. All the identified suspects (from rumor, CBS, and triage) will be tested for COVID-19 by Real-Time Polymerase Chain Reaction (RT-PCR). Negative and positive test findings are intended to be reported first to the federal authority and regional health bureau, where they will be shared with the appropriate Rapid Response Team (RRT) for case management and contact tracing. The RRT will start contact tracing immediately using the contact tracing protocol. Contacts should undergo follow-up for 14 days after the last possible exposure to a confirmed COVID-19 case. If they develop symptoms within 14 days, they will be transferred to an isolation area for further investigation and management [14].

The pandemic is the first of its kind since the country's modern Public Health Emergency Management (PHEM) system was established in 2008, which might put the system's response abilities to the test, especially if the pandemic spreads quickly. The existence of unrecognized cases in the community can be justified by report of cases with unknown contact and cases from a dead body. As of Jun 1, 2020, more than 448 (35.6%) cases were linked to neither travel history nor contact with confirmed cases [15]. This existence of unrecognized cases, combined with the prevailing occurrence of asymptomatic cases, plays a major role in the spread of the disease without targeted control and prevention measures [16].

Given the unprecedented increase in the number of cases, the whereabouts of the cases remain the most important piece of information critical for containing the epidemic. As a result, for the epidemic to be managed, functional epidemiological surveillance system is essential. The two crucial steps to control the spread of the disease, early case detection and isolation, as well as tracing and monitoring their contacts, will be possible only through functional surveillance [17].. The earlier patients and their close contacts are found, the more likely it is to control the development of the epidemic [18]. A functional surveillance system is important to detect and contain outbreaks among vulnerable populations. Surveillance also helps monitor our epidemic response effectiveness in slowing the spread of the epidemic [19]. Furthermore, functional surveillance at local level is important for the global eradication that requires the early detection of new epidemics [20].

Singapore has shown evidence of high sensitivity of case detection in the COVID-19 epidemic [21]. In Hong Kong, containment strategies that include enhanced surveillance and testing have led to control of cases and prevention of a community-wide outbreak during the 4.5 months after the first case was reported [22]. Surveillance has also been used in Colombia to evaluate the effectiveness of COVID-19 control measures [23]. Surveillance data proofed to detect space-time clusters [24].

As a result, it is timely and critical to assess the performance and utility of Ethiopia's COVID 19 surveillance system in containing the epidemic, taking into account both health system-level performance as well as community-level awareness, perceptions, and practices that may influence people's health-seeking behavior. Therefore, we planned to evaluate the early performance of the COVID-19 surveillance system in selected towns of western Oromia, Ethiopia, from August-September 2020. This will help all stakeholders to adjust their activities in a way better achieve the objectives of the COVID-19 surveillance system in their local context.

## Methods

### Study area

The study was conducted in two town administrations called Nekemte and Shambu, Western Ethiopia. Nekemte town is a town administration of East Wollega It is found at East Wollega Zone, Oromia regional state to the west of Ethiopia at a distance of about 328 kilometers from Addis Ababa. It is the center of Western Ethiopia serving as a transient point for different zones and three regional states of the country. Total population of the town is 127,380 among which male constitutes 51.03%. There is one specialized hospital, one referral hospital, two health centers, and seven health posts in the town. Shambu town is the zonal town of Horro Guduru Wollega zone, one of the four zonal towns in Western Oromia. The total population of Shambu town is 24,711 of which 51.8% (12,850) are male. There is one general hospital, one health center, and two health posts providing health services in the town.

### Study design and period

We conducted a community-based and facility-based cross-sectional descriptive study between August and September 2020.

### Study population

All public health facilities and residents of the two towns were the study population.

### Study unit

Surveillance focal person at a different level of the health system and residents of the towns fulfilling inclusion criteria

### Inclusion and exclusion criteria

#### Inclusion criteria

All permanent residents who lived there for at least six months were included in the study.

#### Exclusion criteria

Residents who refused to give us consent were excluded from the study.

### Sample size determination, Sampling procedure, and techniques

**Fig. 2:**
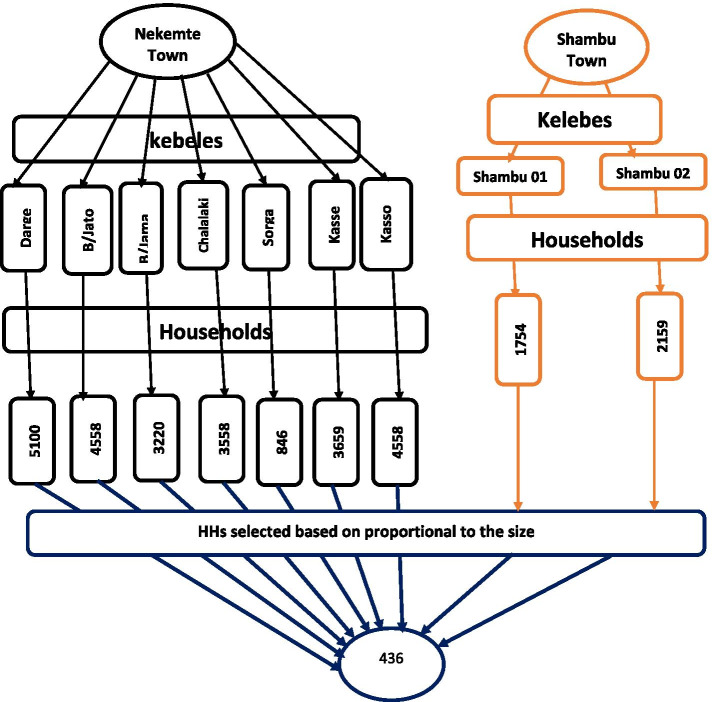
Household selection process based on proportional to size

All health system levels (from health post to specialized hospital) and respective health authorities in the towns were included in the study. The sample size for the community-based study was determined using a single population proportion with assumptions; the prevalence of 50%, 95% confidence interval, and 5% margin of error. Based on the assumptions and adding a 15% non-response rate, the calculated sample size was 441. Systematic random sampling was used to select households. We first determined the number of kebeles in each town and households in each kebele. Then, the sample size was proportionally allocated for each kebele based on the number of households. To determine the households be included in the study, we calculated K by dividing the number of total households by the number of allocated households for that kebele. In each kebele, we have determined the first household by first identifying the center of the kebele and then determining the direction using the lottery method. On the direction identified by the lottery method, data were collected on every k^th^ household until the number of households to be interviewed in the kebele was completed (Fig. 2).

### Data collection methods

#### Community based data collection

To explain community experience and determine what influences their health care usage during this pandemic, a semi-structured questionnaire was used to collect data on community health facility usage (dependent variable) and COVID 19 awareness, practice, socioeconomic status, perceived risk, health extension worker visit, and others (independent variable).

#### Health system based data collection

Data on enhanced IDSR performance for the COVID 19 epidemic was obtained from all health posts, health centers, hospitals, and health office/departments in both towns using an interviewer administered semi structured questionnaire,. The data was collected following the established COVID 19 management protocol [14] and include data on preparedness, suspect identification, rumor gathering, and investigation, testing, and reporting. We used a checklist customized from the CDC surveillance system evaluation guideline to collect data on selected attributes (data quality, simplicity, acceptability, and usefulness) to determine performance in terms of surveillance attributes. We examined reports, suspect and rumor registration books, and data from the house-to-house visit to verify responder reports. We have obtained surveillance data reported to the Regional Health Bureau (RHB) to calculate the positivity rate of samples collected from the group

### Data analysis

We used excel spreadsheet to analyze data from health facilities and health offices/departments. To determine preparedness of health facilities and health offices/departments we have checked for availability of plan, documentation, personnel capacity and enhancement of event based surveillance. This was achieved by simple yes/no options. Data on case detection was described in terms of proportion of suspects tested and turned positive through house to house visit by health extension workers and triage at health facilities. Performance of surveillance in terms of selected surveillance attributes (data quality, simplicity, acceptability, and usefulness) was described in terms of proportion of respondents asserted for good level of individual attribute.

In addition, community data was entered into epi data 3.1 and then exported to the Statistical Package for the Social Sciences (SPSS) software window version 24 for analysis. Proportions, mean, and standard deviation were used to describe community data. After that, bivariable analysis was used to see if there was any association between the dependent and independent variables. To find independent predictors, all variables with a PV of 0.25 were included in the final model of multivariable logistic regression. Variables maintained association at a p-value of 0.05 (Pv<0.05) significance level were considered independent predictors for not using health facility during this pandemic. We checked the model goodness of fit by Hosmer and the lemeshow test. Finally, we discussed how the community's experience during the pandemic, as well as current health-care practices, could affect COVID 19 surveillance performance.

## Results

### Health system

#### Preparedness

Two hospitals, three health centers, one town health office, one zonal health department, and nine health posts have participated in the study. Only 1of 15 (6.7%) organizations assessed have guidelines/manuals of COVID-19 surveillance. All health centers, hospitals, and health offices were trained on the COVID-19 surveillance system, while 4 (44%) of the interviewed health extension workers reported that they did not get training. Rapid Response Team (RRT) has been established at all levels in the towns. The updated case definition was found in 14/15 (93.3%) of the visited facilities.

In both towns, preparedness about implementing surveillance systems from plan to community engagement is presented as follows (Table 1).

**Table 1:** Preparedness concerning COVID-19 surveillance system implementation, of selected towns’ health system, West Oromia, Ethiopia, Aug 2020

Towns	Organization	Guideline	Plan	Training	CD	Established RRT	Conducted RA	Conducted RC	Identified Vulnerable group	Established CBS	Organized Volunteers
Nekemte Town	Health office	Y	Y	Y		Y	N	N	N	Y	
	Hospital	N	Y	Y	Y	Y	Y	Y			
	Health center	N	Y	Y	Y	Y	Y	Y			
	HP1	N		Y	Y						N
	HP2	N		N	Y						Y
	HP3	N		N	Y						N
	HP4	N		N	Y						Y
	HP5	N		N	Y						N
	HP6	N		Y	Y						Y
	HP7	N		Y	Y						Y
Shambu Town	Zone health department	N	Y	Y		Y	Y	Y	N	Y	
	Hospital	N	N	Y	N	Y	N	N			
	Health Center	N	Y	Y	Y	Y	Y	Y			
	HP1	N		Y	Y						Y
	HP2	N		Y	Y						Y

Key: CD: Case definition, RRT: Rapid Response Team, RA: Risk Assessment, RC: Risk communication, CBS: Community Based Surveillance, HP: Health Post

### Suspect identification and reporting

#### Suspect from house to house visit

A house-to-house visit for risk group identification and suspect identification was done only between April and May 2020. Between these periods, health extension workers have visited 12,012 of 25,413 (47.2%) households in Nekemte town and identified 7 suspects, of which 3 became positive for COVID-19. In Shambu town, 3,012 of 3,931 (76.9%) households have been visited, but no data were available that indicate the number of suspects identified and tested.

#### Suspect from triage

At the facility level, there was no screening service at triage in both towns. The available service for screening for COVID-19 was the outpatient department. Total of 173 suspects have been identified from Nekemte town, none of which were tested positive. In Shambu town, 144 suspects were identified, four of which tested positive (Table 2).

**Table 2:** Suspect identification and testing by health system level, COVID-19 surveillance system evaluation, Western Oromia, Ethiopia, Aug 2020.

Level	Town	Suspect identified	Suspect Tested	Result			
				Positive	Negative	Pending	Blank
Health post	Nekemte	7	7	3	4	0	0
	Shambu	Data unavailable					
Health facility	Nekemte	173	173	0	170	3	0
	Shambu	144	144	4	140	0	0

No rumor and cluster investigation were practiced at both towns.

#### Case detection and reporting

Once suspect was detected, the Rapid Response Team (RRT) has been notified to communicate with Nekemte Public Health Research and Referral Laboratory (NPHRRL) or Wollega University Referral Hospital for sample collection and testing. The results were communicated to the Regional Health Bureau and the Federal Ministry of Health, Ethiopian Public Health Institute (EPHI). The Regional Health Bureau is responsible for communicating results back to the RRT for necessary action.

### Surveillance attributes

#### Data quality

There was no consistency in reporting of the date of testing, either Gregorian or Ethiopian calendar was in use. Individuals suspected of COVID-19 can be tested several times without being recognized, as there is no control mechanism for repeated testing except for those under treatment.

#### Simplicity

Of the visited health facilities, 75% considered variables on the report are simple to understand. Half (50%) of the health facilities reported that means of data collection are convenient for them.

#### Acceptability

More than half, 75% of the health facilities in both towns reported that they have a complaint about the surveillance system. The main reasons for complaint were lack of support from authority (100%), many reportable variables (67%), and internet interruption (67%).

#### System usefulness

Laboratory-based surveillance was being used for contact tracing and case management. Data analysis was not being done at both towns to identify the most at-risk group and location. The health authorities in both towns are not using surveillance data for decision-making.

### Community Based Assessment

#### Characteristics of respondents

Four hundred thirty-six study participants were involved in the analysis with a response rate of 98.9%. The mean age of the study participants was 37.87 with a standard deviation (SD) of 12.5 with 192 (44%) females. More than half (58.5%) was Protestant followed by Orthodox and Muslim constituting 25% and 15.4% respectively. More than three-fourth (83.5%) were married. Hundred seventy-seven (40.6%) of them achieved college and above education level. Nearly one quarter (32.6%) of the total study participants was government and/or nongovernmental organizations’ employees and 157 (36.0%) of them rely on their salary to live.

#### Community risk assessment and awareness

There were high-risk individuals in 115 (26.4%) of the visited households (either age or underline medical condition). One hundred seventy-nine (41.1%) of the participants perceived they have a high risk of contracting COVID-19. Four hundred two (92.2%) of the respondent knows how to protect oneself from COVID-19. Four hundred twenty (96.3%) of the respondent reported they get information about COVID-19 from TV/radio (Table 3).

**Table 3:** Community risk and awareness assessment among dwellers of selected towns, west Oromia, Ethiopia, Aug 2020, (n=436)

Variables and description		Number	%
Household (HH) members traveling from place to place	Yes	109	25.0
	No	327	75.0
Presence of healthcare workers in the HH	Yes	40	9.2
	No	396	90.8
Presence of highly mobile individual in the HH	Yes	135	31.0
	No	301	69.0
High-risk individuals in HHs	Yes	115	26.4
	No	321	73.6
Perceived level of risk of contracting COVID-19	High	179	41.1
	Medium	107	24.5
	Low	134	31.7
	Do not Know	16	3.7
Visited by health extension workers (HEWs)	Yes	127	29.1
	No	309	70.9
Frequency of visit by HEW	Once	77	60.6
	Twice	38	29.9
	Three or more times	12	9.4
Ever heard about COVID-19	Yes	428	98.2
	No	8	1.8
Source of information about COVID-19	Television/radio	420	96.3
	Social media	271	62.2
	Health professionals	208	47.7
	Religious leaders	141	32.3
	Community leaders	129	29.6
	Family members	98	22.5
What do you know about COVID-19;	Do not know anything	38	8.7
	It’s a virus that can cause a disease	389	89.2
	It’s a government’s program	2	0.5
	It’s a TV/radio campaign	2	0.5
	Other*	5	1.1
Information gained	Protection methods	402	92.2
	Symptoms	384	88.1
	Transmission ways	366	83.9
	Actions to be taken when contract COVID-19	258	59.2
How dangerous COVID-19 is;	Very dangerous	353	81.0
	Almost dangerous	72	16.5
	Is not dangerous	7	1.5
	Other**	4	0.9
Possibility to be infected by COVID-19	Yes	287	65.8
	No	122	28.0
	Don´t know	27	6.2

Key: Other * *(it is the curse of God, is an intentional product from other countries),* other** *(it is fatal, it comes and go)*

#### Community experience and practices

**Fig. 3:**
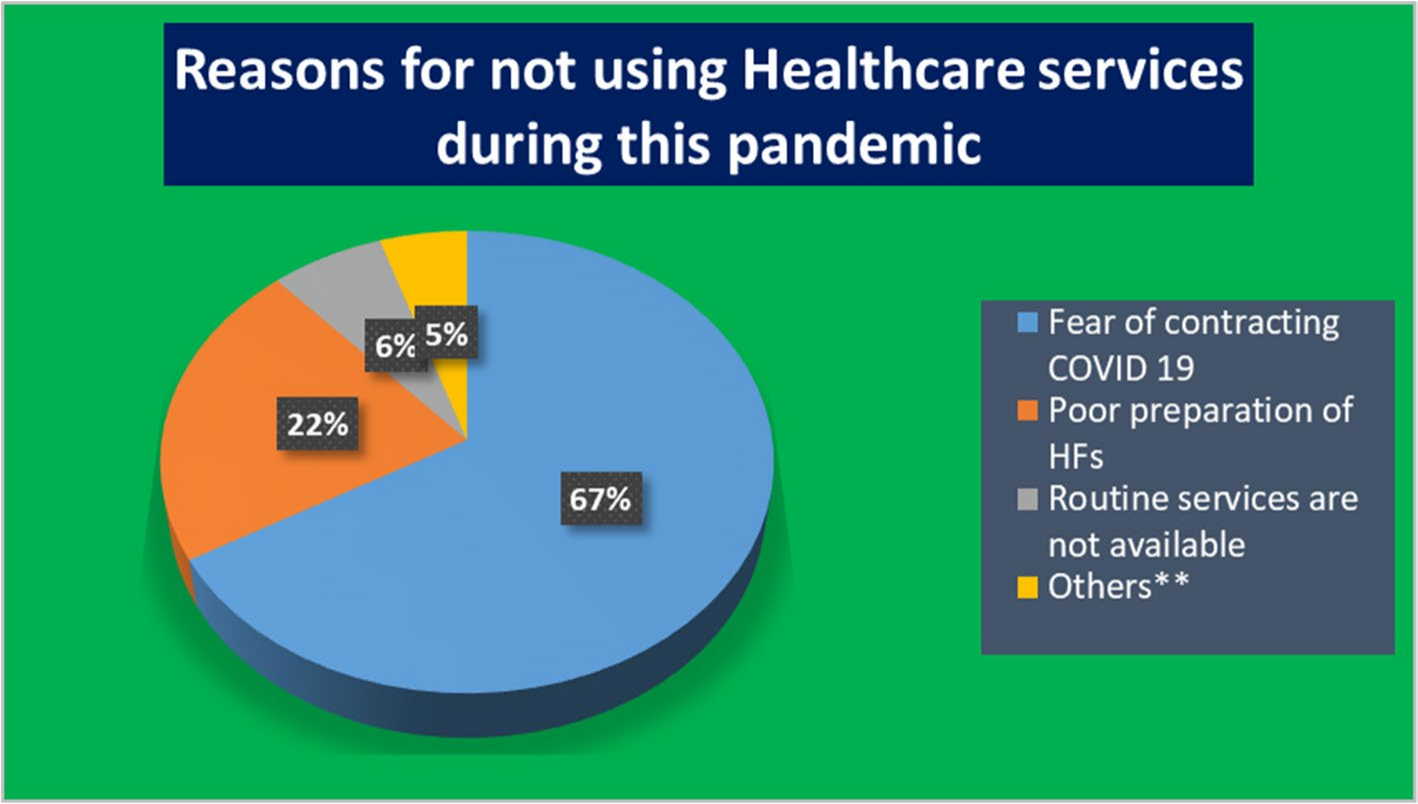
Reasons for not using routine healthcare services during COVID-19, Western Oromia, Ethiopia (n=197). Others** include: prefer to be at home, healthcare professionals are uncooperative, not get sick

Four hundred twenty-five (97.5%) of the participants reported that they will report to the health system if they suspect an individual for COVID-19. From the total visited households, 182 (41.7%) reported that their healthcare visiting experience has been affected by the emergence of COVID-19. One hundred ninety-seven (45.2%) reported that they were not using health facilities for routine services during this pandemic (Table 4). Among those who reported not using health facilities during this pandemic, 132 (66.7%) were not using health facilities because they fear contracting COVID-19 (Fig. 3).

**Table 4:** Community experience and practice assessment toward COVID-19 in selected towns, west Oromia, Ethiopia, Aug 2020, (n=436)

Variables	Description	Number	%
Experience during feeling ill health	Visit health facility	360	82.6
	Go to pray	47	10.8
	Purchase some medicine	23	5.3
	Eat foods believed to be a remedy	4	0.9
	Other^*^	2	0.5
Access to health services affected by this pandemic	Yes	182	41.7
	No	254	58.3
Using health facilities during the pandemic	Yes	239	54.8
	No	197	45.2
Willing to be isolated if contract COVID-19	Yes	409	93.8
	No	27	6.2
Practices if contract COVID-19	I will take locally advised foods, garlic, ginger, soups, etc.	227	52.1
	I will go to the hospital/health unit	400	91.7
	I will go to the neighborhood nurse	46	10.7
	I will buy medicines at the market	19	4.4
	I will look for the traditional healer	12	2.8
	I would stay in quarantine	161	36.9
Action to be taken on suspecting person with COVID-19 symptoms	Report to local health	256	58.7
	Call 8335 or local call center	169	38.8
	Do not know or Do nothing	11	2.5
heard someone died of COVID-19	Yes	13	3.0
	No	423	97.0
Heard neighbor died of COVID-19	Yes	9	2.1
	No	427	97.9

Key: others^*^, include do nothing, stay at home

#### Community health facility usage during this pandemic and associated factor

On multivariable logistic regression analysis, the source of income AOR=0.30, 95% CI (0.11, 0.86), perceived level of risk AOR=3.42, 95% CI (2.04, 5.7) and being visited by health extension workers AOR=0.46, 95% CI (0.29, 0.74) were found to be independent predictors of not using health facilities during this pandemic (Table 5).

**Table 5:** Factors associated with health facility usage during this pandemic, cross-sectional study, West Oromia, 2020

Independent Variable	Category	HF usage during this pandemic		COR	AOR	PV
		No n (%)	Yes n (%)			
Age group	18–24	19(43.2)	25 (56.8)	1		
	25–34	66 (46.2)	77(53.8)	0.89 (0.45, 1.75)		
	35–44	62 (46.3)	72 (53.7)	0.83 (0.44, 1.75)		
	45–54	31 (48.4)	33 (51.6)	0.81 (0.02, 5.76)		
	≥55	19 (37.3)	32 (62.7)	2.48 (0.75, 8.28)		
Sex	Male	116 (47.5)	128 (52.5)	1		
	Female	81 (42.2)	111 (57.8)	1.24 (0.85, 1.82)		
Marital status	Single	20 (45.5)	24 (54.5)	1		
	Married	165 (45.3)	199 (54.7)	1.00 (0.54, 1.88)		
	Divorced	6 (43.5)	5 (54.5)	0.69 (0.18, 2.62)		
	Widowed	6 (35.3)	11 (64.7)	1.53 (0.48, 4.87)		
Educational status	illiterate	15 (44.1)	19 (55.9)	1		
	Read and write or elementary	43 (41.7)	60 (58.3)	1.1 (0.5, 2.41)		
	High school and above	139 (46.5)	160 (53.5)	0.91 (0.45, 1.86)		
Source of income	Salary (Gov/NGO	75 (38.1)	82 (52.2)	1	1	
	Petty business	63 (36.4)	110 (63.6)	0.98 (0.0006, 1.72)	0.95 (0.34, 2.80)	0.91
	Large business	21 (51.2)	20 (48.8)	0.08 (0.004, 1.73)	0.68 (0.20, 2.70)	0.46
	Day laborer or jobless	38 (58.5)	27 (41.5)	**0.01 (0.00, 0.40)**	**0.30 (0.11, 0.86)**	**0.025**
Presence of health professional in the house	Yes	20 (50)	20 (50)	1		
	No	177 (44.7)	219 (55.3)	1.24 (0.65, 2.37)		
Presence of a high-risk person in the house	Yes, elder	40 (47.6)	44 (52.5)	1		
	Yes, chronic	13 (41.9)	18 (58.1)	1.26 (0.55, 2.89)		
	No	144 (44.9)	177 (55.1)	1.12 (0.69, 1.81)		
Perceived level of risk	High	98 (54.7)	81 (45.3)	1	1	
	Medium	51 (47.7)	56 (52.3)	1.33 (0.82, 2.15)	1.46 (0.87, 2.55)	0.15
	Low	38 (28.4)	96 (71.6)	**3.06 (1.20, 4.93)**	**3.42 (2.04, 5.7)**	**0.00**
	Do not know	10 (62.5)	6 (37.5)	0.73 (0.25, 2.08)	1.12 (0.37, 3.40)	
Visited by health extension worker (HEW)	Yes	45 (35.4)	82 (64.6)	1	1	
	No	152 (49.2)	157 (50.8)	**0.57 (0.37, 0.87)**	**0.46 (0.29, 0.74)**	**0.001**
How dangerous is COVID-19?	Very dangerous	155 (43.9)	198 (56.1)	1		
	Almost dangerous	35 (48.6)	37 (54.4)	0.83 (0.50, 1.38)		
	Not dangerous and other	7 (63.6)	4 (36.4)	0.45 (0.13, 1.56)		

### Discussion

In this study, we found that risk group testing detected more cases than suspect or contact testing within the first five months of enhanced COVID-19 surveillance implementation. During epidemic management, the major goal of surveillance is to find cases early in order to reduce transmission. This is possible by identifying more suspects, targeting at-risk groups, and monitoring vulnerable groups. From a system perspective, public health surveillance data are the result of a series of decisions made by patients about seeking health care and reporting rumors, healthcare providers about providing health care, and public health professionals about reporting cases or otherwise taking actions that come to the attention of health authorities.

The engagement of local community was expected to enhance suspect identification through event based surveillance approach. Health offices from both towns had set up Community Based Surveillance (CBS), but one-third of the health posts (all from Nekemte town) didn't have any community volunteers who could have assisted them with suspect identification. The lack of community volunteers in majority of health posts in Nekemte Town may have contributed to the low case detection performance. From the Regional Health Bureau laboratory-based surveillance data, up to July 30, 2020, there were 150 contacts, 115 suspects, and 521 risk groups tested for COVID-19-from Nekemte town. A positive result was found only from the risk group testing, 10/521 (2%).

Furthermore, majority of the communities are aware about the disease: cause (96%), transmission (83.9%), symptoms (88.1%), protection methods (92.2%) and action to be taken if get infected (59.9%) mainly obtained from TV/radio (96.3%). In contrary, in Jordan and Iraq major source of COVID-19 related information was medical staff (60%) [25]. This difference could be due to method used for data collection, which was online platform in the study done in Jordan and Iraq. Awareness about the disease is important to implement the recommended prevention measures [26, 27]. In addition, this community could have been used for early case detection, as part of community based surveillance. However, there is no link between the community and the health system that enables the community to report any rumor in their locality. As a result, health extension workers in Nekemte town identified just seven suspects for the two months they have been doing house to house visits, while there were no data for Shambu town. Community participation in screening and follow-up is critical for surveillance performance [20]. Community event based surveillance have improved early outbreak case detection in pilot implementation in Vietnam [28], detected 30% of the Ebola Virus Disease (EVD) cases identified and majority of cases with no epidemiological links in Sierra Leone [29] and described as useful in detecting a large scope of events, reaching remote areas and guiding outbreak response in systematic review of event based surveillances in low and middle income countries [30]. Furthermore, community health volunteers played critical role in Thailand’s effective COVID 19 management [31].

The risk group screening was the primary source of cases in this investigation. This reflects the extent to which the epidemic has spread throughout the community. Furthermore, 45.2 percent of respondents said they are not using health facilities for routine services because of the pandemic. Hence, less likely to get captured by suspect identification system. In a study done in Wuhan, China, only 35% of people with acute respiratory infection visited health care during the outbreak of novel coronavirus disease [32]. This finding is opposite to the finding of Xiaoli Wang et al. in Beijing, whereby the proportion of healthcare usage of a general hospital during an epidemic period is higher than that was during the non-epidemic period for influenza [33]. Severity of the disease can affect health care-seeking behavior [34]. In this study, the odds of not using health care in jobless/day laborers were 70% lower than those having salary (Government or NGO) (PV=0.025), this is contrary to a study done in the UK during the 2009/2010 A/H1N1 influenza pandemic [35]. The odds of low perceived risk were 3.42 times greater among those who were not using health care during this pandemic than who were using (PV=0.00). Different amounts of information about the pathogen, quality of local healthcare, availability of preventive measures, and individual and group use of intuition in the decision-making process could all contribute to the disparity in risk perception that may affect health care seeking behavior [36]. The odds of not using healthcare in those not visited by health extension worker were 54% lower than those visited (PV=0.001). This was because persons who were visited by health extension workers were given advice on how to behave during the epidemic and were more aware of the use of visiting healthcare facilities, regardless of the COVID 19 report coming out of the health facilities.

However, in the early study of patients in Wuhan, China, contact tracing contributed to the primary detection of approximately half (53%) of COVID-19 patients [37]. This can be explained by the early situation during the epidemic when people may not be aware of most of the prevention measures and have a social gathering where a single infectious individual can infect a dozen susceptible individuals. Almost all the individuals in charge of running the surveillance are trained and are comfortable with the interpretation of reportable variables. Nonetheless, three forth of them have a complaint about surveillance system management. Their main concerns are a lack of support from an authority, many reportable forms and variables, and internet interruption. These may contribute to the observed data discrepancy across the health system level.

Epidemic control requires knowing trends in disease frequency in different subgroups and locations. A surveillance system for COVID-19 is essential to understand the burden across the different strata of any health system and the population [38]. However, in our study, we found that surveillance data are not analyzed and hence, not being used to identify the most at-risk group and location and to monitor the outcome of response activities in the local context. Furthermore, surveillance data analysis is important to identify a cluster of cases to which leads to cluster investigation for the sake of understanding the main route of transmission in the local setting for containing locally acquired cases are critical to prevent widespread community transmission [39, 40].

Even though this study is the first of its kind to evaluate the surveillance system of COVID-19 in the study area, we did not assess the actual prevalence of the disease in the community to compare the exact performance of the surveillance with the actual prevalence in the community.

## Conclusions

In conclusion, event based surveillance, both at community and health facility level, was not performing optimally in identifying and linking potential suspects for further investigation in the study area. In addition, the link between the community and health system was poor, though community awareness of suspect identification and reporting is good. Subsequently, sensitivity of community/risk group testing was higher than that of suspect testing. Surveillance data were not being used to identify the group and area most exposed for guiding response strategy. The effectiveness of routine facility-based surveillance will be as good as the community's health facility usage. Therefore, for effective early containment of epidemic, it is critical to strengthen event based surveillance and make use of surveillance generated information as a guide for tailored intervention in settings where mass testing is not feasible. Appropriate community engagement and support to encourage presentation and compliance are essential. The authorities’ continuous support is also highly recommended for better performance of surveillance data quality.

### List of acronyms/abbreviations

CBSCommunity-Based Surveillance

CDCCenters for Disease Control and Prevention

COVID-19Coronavirus Disease 2019

FMOHFederal Ministry of Health

HEWHealth Extension Worker

PHEMPublic Health Emergency Management

RHBRegional Health Bureau

RRTRapid Response Team

RT PCR Real-Time Polymerase Chain Reaction

SARS-CoV-2Severe Acute Respiratory Syndrome coronavirus 2

SDStandard Deviation

SPSSStatistical Package for the Social Sciences

TVTelevision

WHOWorld Health Organization

## Data Availability

The data sets used and analyzed during the current study are available from the corresponding author on reasonable request.
